# Evaluation of functional outcomes and preliminary results in a case series of 15 children treated with arthroscopic release for internal rotation contracture of the shoulder joint after Erb’s palsy

**DOI:** 10.1007/s11832-016-0773-1

**Published:** 2016-10-12

**Authors:** Mohamed Mansour Elzohairy, Adel Mohamed Salama

**Affiliations:** 1 Orthopaedic Surgery department, Faculty of Medicine Zagazig University, Zagazig, Egypt; 2 Dr. Mansour Elzohairy Villas, 4. Zeid Ben Sabet Street, University Villas, Zagazig, Sharkia Governorate Egypt

**Keywords:** Arthroscopic, Release, Internal rotation, Contracture, Shoulder joint, Erb’s palsy

## Abstract

**Introduction:**

The purpose of this study was to evaluate shoulder function following minimally invasive subtotal subscapularis muscle and periarticular capsuloligamentous arthroscopic release in children with Erb’s palsy.

**Methods:**

A prospective study was conducted on 15 consecutive children who underwent subtotal subscapularis muscle and periarticular capsuloligamentous arthroscopic release to treat internal rotation contracture of the shoulder joint after Erb’s palsy. Age at surgery ranged from 24 to 38 months (average 28.3) (2.4 years). All of the patients were assessed clinically and radiologically preoperatively and postoperatively at regular intervals. The Mallet scoring system was used to analyze the results.

**Results:**

The mean external rotation improved from −24° to +46° (*p* = 0.001) at the last follow-up. Active internal rotation was preserved in all cases. At the final follow-up, there had been no loss of the external rotation gained and no recurrence of internal rotation contracture of the shoulder, and the mean Mallet score (total) had improved from 11 to 17 points (*p* = 0.001).

**Conclusions:**

In children aged from 1 to 3 years, an arthroscopic release procedure alone may successfully restore function and yield a centered glenohumeral joint, which has a beneficial effect on glenoid remodeling.

**Level of evidence:**

Level IV.

**Electronic supplementary material:**

The online version of this article (doi:10.1007/s11832-016-0773-1) contains supplementary material, which is available to authorized users.

## Introduction

Shoulder dysfunction in infants who suffer brachial plexus injuries as a result of C5/C6 upper trunk lesions leads to an imbalance between the medial rotators of the shoulder (which are relatively unaffected) and the lateral rotators of the shoulder [1–3]. Therefore, associated internal rotation myostatic contractures of the subscapularis and pectoralis major with significant periarticular capsuloligamentous tightness can often develop, which require release [2, 4]. These soft-tissue imbalances around the glenohumeral articulation, if left untreated, will result in secondary dislocation or subluxation and a fixed articular deformity of both the humeral head and the glenoid with overgrowth of the coracoid [5]. Recently, arthroscopic release has been performed in young children for the anterior glenohumeral ligaments, capsule, and upper intra-articular subscapularis tendon, with or without tendon transfer(s), to restore external rotation and abduction of the shoulder and subsequently improve the morphology of the glenohumeral joint [6]. The aim of the present study was to evaluate minimally invasive subtotal subscapularis muscle and periarticular capsuloligamentous arthroscopic release alone (i.e., without tendon transfer) for the treatment of internal rotation contracture of the shoulder joint due to Erb’s palsy, and to compare this procedure with other relevant methods reported in the literature. The Mallet score was used to evaluate functional outcome [7, 8].

## Materials and methods

This prospective study was conducted from January 2009 to December 2014. Fifteen patients (9 boys and 6 girls) were operated on for internal rotation contracture of the shoulder joint due to residual Erb’s palsy. The average age of the children at surgery was 28.3 (24.2–38.1) months (2.4 years). Clinical follow-up was carried out monthly in all children, which involved recording the range of movement for both shoulder joints. Combined abduction and anterior flexion was measured while the child was playing. Rotation was evaluated based only on passive movements as it is difficult to quantify active movements in children. Passive external rotation was measured preoperatively and postoperatively with the arm by the side of the body. The Mallet classification score was used to assess upper extremity function in children with brachial plexus birth palsy (Table 1) [7, 8]. A rapid loss of passive external rotation between monthly examinations is indicative of progressive capsular and muscular contracture and the subsequent onset of subluxation or dislocation. The main indication for the surgical procedure was a passive external rotation of ≤0°. The second indication for it was an age of 1–3 years.

**Table 1:** Measurement of internal rotation by the Mallet score (functional parameter)

Hand to great trochanter	1 point
Hand to buttocks	2 points
Hand to L4	3 points
Hand to T12	4 points
Hand to T6 (normal)	5 points

### Inclusion/exclusion criteria

Inclusion criteria were: children from 12 to 38 months (1–3.2 years) of age; unilateral; involvement of the C5, C6, and C7 nerve roots; patients received functional rehabilitation from 3 weeks of life and active and effective flexion of the elbow was recovered at between 3 and 6 months of age; and patients did not undergo surgery of the nerves or plexus (either neurolysis or neurotization) before or after the arthroscopic release procedure. Other patients who were above or below the specified age range or did not meet these criteria were excluded from the study. All surgeries were performed by the authors of this paper.

### Operative technique

**Fig. 1:**
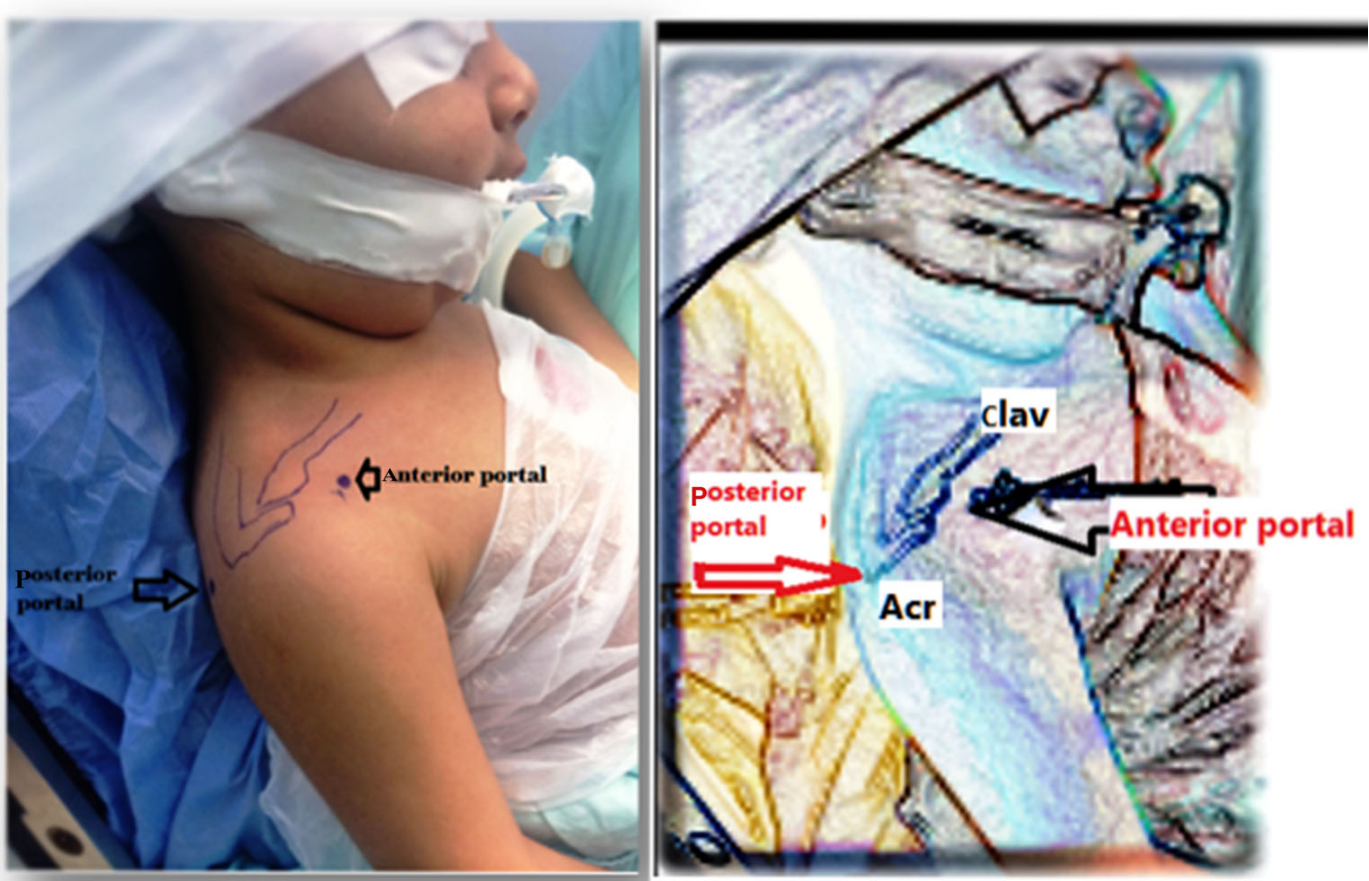
Anatomical landmarks with anterior and posterior portal identification; *clav* clavicle, *Acr* acromion

**Fig. 2:**
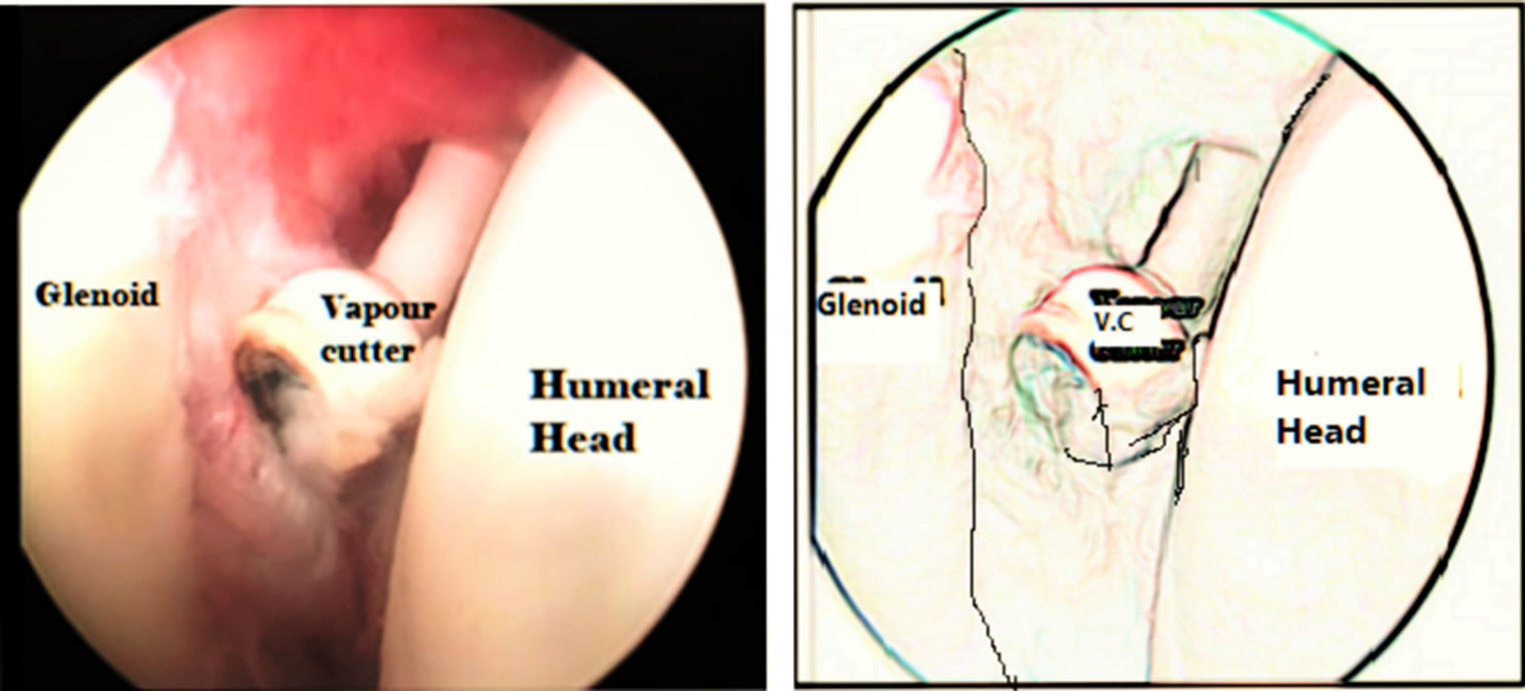
An electrocautery vapor was introduced through the anterior portal; *VC* vapor cutter

**Fig. 3:**
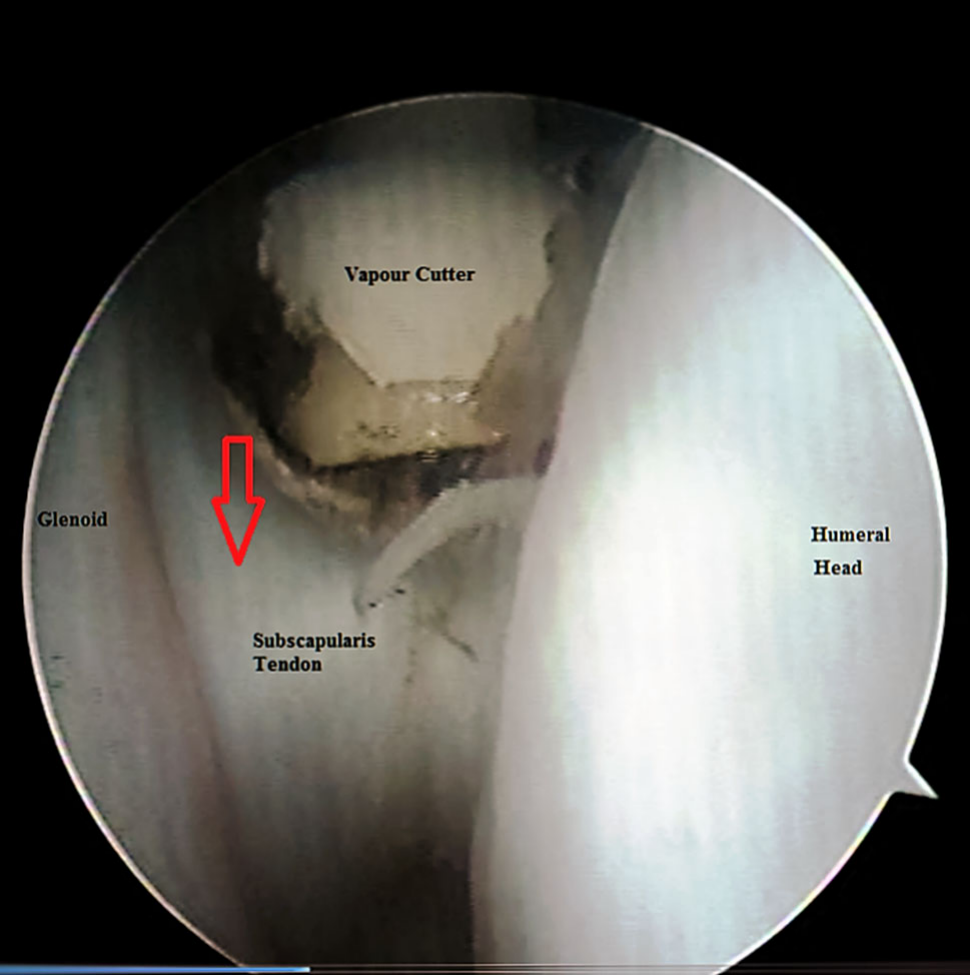
The subscapularis tendon to its muscular portion was identified, and release was continued solely to the capsule, taking care to preserve the inferior and lateral portions of the subscapularis tendon

**Fig. 4:**
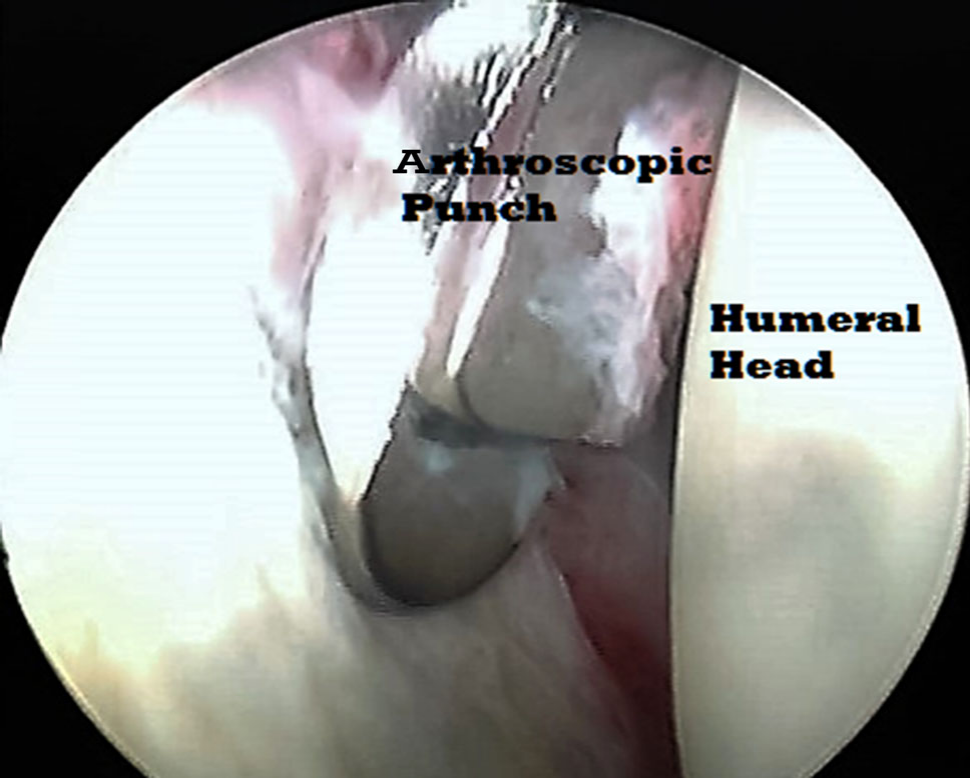
An arthroscopic punch was then used to release the inferior glenohumeral ligament

Under general anaesthesia, and without any traction apparatus, passive mobility was reassessed in the supine beach chair position. Passive external rotation (with the arm at the side and at 90° of abduction) and passive abduction of the shoulder were evaluated. After the landmarks had been identified (Fig. 1), a small-joint 2.7 mm 30° short arthroscope was used in all procedures. Using a 20G spinal needle, the glenohumeral joint was distended with about 20 ml of saline. Taking care not to go too low (to avoid injury to the articular surface), the posterior portal was created at the posterolateral corner of the acromion. The assistant held the arm at approximately 90° of abduction while applying longitudinal traction to overcome the joint contracture and to facilitate the entry of the arthroscope into the joint through the posterior portal. With the aid of a spinal needle, the anterior portal was arthroscopically visualized from the posterior portal. The anterior capsule, anterior glenohumeral ligaments, rotator interval, and subscapularis tendon were identified. An electrocautery vapor was introduced through the anterior portal (Fig. 2). The thickened superior and middle glenohumeral ligaments along with the upper intra-articular portion of the subscapularis tendon were released. Then the transition of the subscapularis tendon to its muscular portion was identified, and the release was continued to the capsule, taking care to preserve the inferior and lateral portions of the subscapularis tendon to maintain the active internal rotation (Fig. 3). An arthroscopic punch was then used to release the inferior glenohumeral ligament, taking care not to injure the axillary nerve (Fig. 4). Eventually, if passive external rotation of >70° was obtained, no additional release of the subscapularis tendon or the axillary pouch was deemed necessary. The postoperative immobilization was by shoulder spica cast at 90° of abduction and 70° of external rotation for 4 weeks. As soon as the plaster was removed, the rehabilitation program was started (see the video in the Electronic supplementary material, ESM).

### Statistical analysis

SPSS for Windows, version 16.0 (Chicago, IL, USA, 2007) was used for data analysis, and *p* > 0.05 was considered to indicate statistical significance.

The Wilcoxon signed-rank test was used to compare preoperative with final follow-up values in the present study.

## Results

**Fig. 5:**
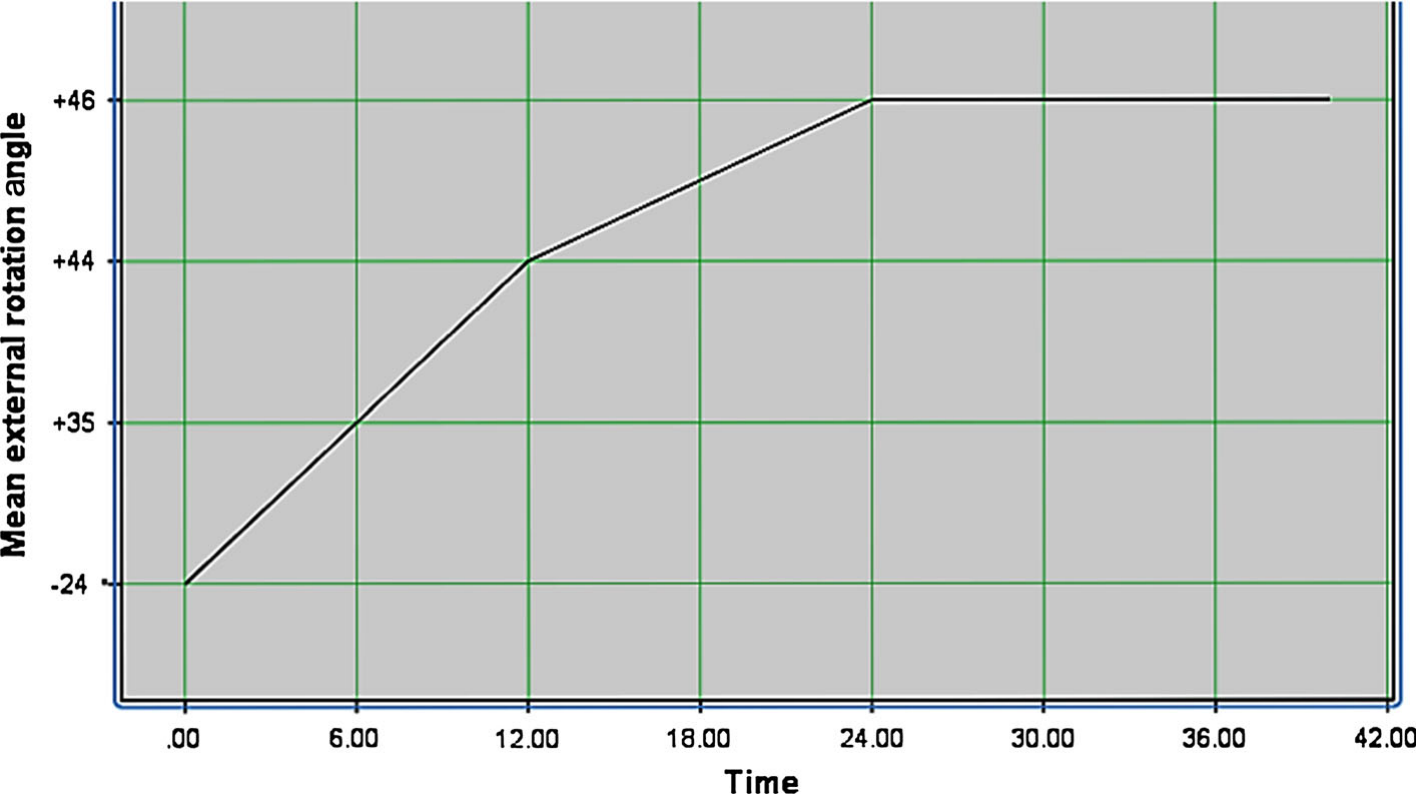
Evolution of external rotation with time (in months)

**Fig. 6:**
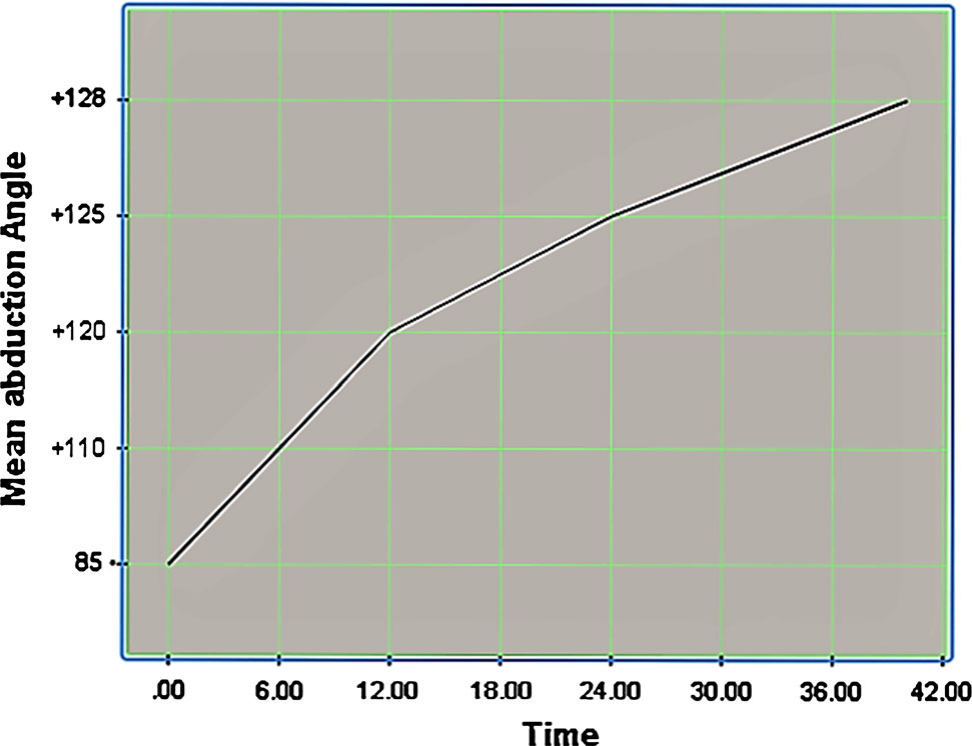
Evolution of abduction with time (in months)

**Fig. 7:**
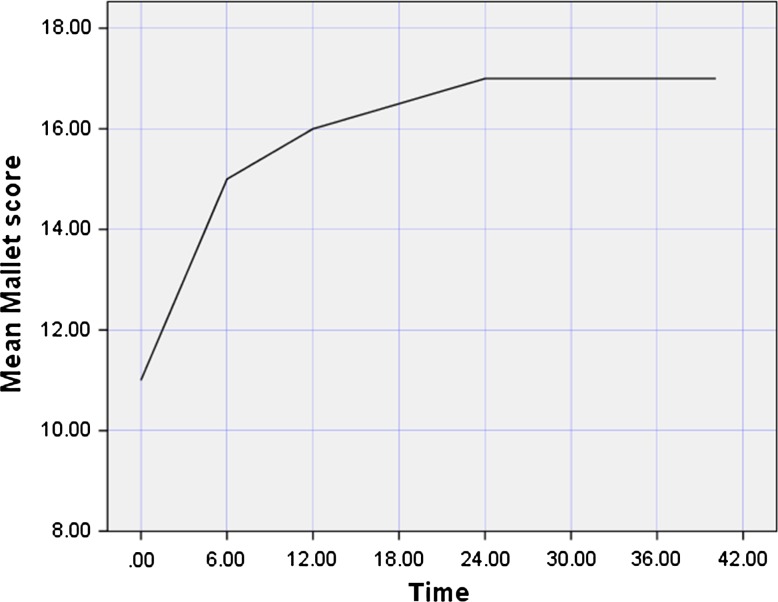
Evolution of Mallet score with time (in months)

The children were followed up for periods ranging from 26 to 59 (average 40.1) months (3.3 years). All cases were unilateral. In eleven of them, the C5 and C6 nerve roots were involved, while the C5, C6, and C7 nerve roots were involved in the other four. The mean parameters—the mean external rotation, the mean elevation, and the mean Mallet score (total)—were improved at the last follow-up. The mean percentage of the humeral head anterior to the middle of the glenoid fossa (PHHA) and the mean glenoid retroversion were improved at the last follow-up. No complications were observed in this series. Regarding the learning curve, the mean operative time decreased from an average of 60 to 37 min for the last case to be operated on, including patient positioning. There was no recurrence of the internal rotation contracture at the last follow-up (see Figs. 5, 6, and 7 and Tables 2 and 3).

**Table 2:** The mean pre and postoperative last follow-up, angles and parameters

	M.Pre.Ext.Ro.	M.Post.Ext.Ro.	M.Pre.E.	M.Post.E.	M.Pre. (PHHA)	M.Post. (PHHA)	M.Pre. GRV	M.Post. GRV	M.Pre.M.S.	M.Post.M.S.
Mean value	−24°	+46°	85°	128°	15 %	45 %	−34°	−18°	11	17
*p*	0.001*		0.001*		0.001*		0.001*		0.001*	

*M.Pre.Ex.Ro.* mean preoperative external rotation, *M.Post.Ex.Ro.* mean postoperative external rotation, *M.Pre.E.* mean preoperative elevation, *M.Post.E.* mean postoperative elevation, *Pre. PHHA* mean preoperative percentage of the humeral head anterior to the middle of the glenoid fossa, *Post. PHHA* mean postoperative percentage of the humeral head anterior to the middle of the glenoid fossa, *M.Pre. GRV* mean preoperative glenoid retroversion, *M.Post. GRV* mean postoperative glenoid retroversion, *M.Pre.M.S.* mean preoperative Mallet score (total), *M.Post.M.S.* mean postoperative Mallet score (total),

* *p* > 0.05 or was considered to indicate a statistically significant difference

**Table 3:** Summary of the results of applying the present technique, and comparison of that technique with similar and other relevant surgical techniques reported in the last 10–15 years

Study	Number of patients included	Type of surgery	Age (mean, years)	FU (years)	PHHA (%)		Glenoid retroversion (°)	
					Preop.	Postop.	Preop.	Postop.
Kozin et al. [24]	44	Arthroscopic release (*n* = 28); arthroscopic release/tendon transfer (*n* = 16)	2.7	1	19 ± 12	33 ± 12	−34 ± 15	−19 ± 13
Mehlman et al. [20]	50	Arthroscopic release (*n* = 34); arthroscopic release/tendon transfer (*n* = 16)	5.1	2	30.5	38.8	−25	−14.1
Pearl et al. [22]	33	Arthroscopic release only (*n* = 15)	1.4	2	–	–	–	–
		Arthroscopic release/late tendon transfer (*n* = 4)	1.8		–	–	–	–
		Arthroscopic release/tendon transfer (*n* = 14)	6.7		–	–	–	–
Pedowitz et al. [23]	22	Arthroscopic release (*n* = 7); arthroscopic release/tendon transfer (*n* = 15)	3.9	1 month (postop; imaging in spica cast)	15.6 ± 13.5	46.9 ± 11.2	−37 ± 2	−8 ± 8
Kany et al. [4]	16	Arthroscopic release/tendon transfer (*n* = 16)	1.7	3.5	–	–	–	–
Kokkalis et al. [6]	1	Arthroscopic release (*n* = 1)	2.5	1.5	–	–	–	–
Naoum et al. [25]	64	Carlioz proximal subscapularis release (*n* = 64)	2.6	4.2	–	–	–	–
Current study	15	Arthroscopic release (*n* = 15)	2.4	3.3	15 %	45 %	−34°	−18°

Study	Deformity		External rotation (°)		Elevation (°)		Mallet score (total)	
	Preop.	Postop.	Preop.	Postop.	Preop.	Postop.	Preop.	Postop.
Kozin et al. [24]	2.9 ± 1.0	1.9 ± 0.4	−26 ± 20	47 ± 17	112 ± 28	130 ± 38	12.7 ± 1.6	17.1 ± 1.4
Mehlman et al. [20]	2.8	1.9	–	–	–	–	12.6	16.3
Pearl et al. [22]	Marked remodeling in 12 of 15 children with a pseudoglenoid deformity		−2	64	94	106	–	–
			−15	62	115	125	–	–
			−24	56	121	124	–	–
Pedowitz et al. [23]	–	–	–	–	–	–	–	–
Kany et al. [4]	–	–	The average preoperative external rotation was −11.5° (0–20°), the internal rotation according to Mallet’s score was on average 2.3 points, and the average abduction and forward flexion was +62° (50–90°). In the immediate postoperative period, the passive external rotation was +65.5° (60–70°), with an average gain of +77° (70–90°). On follow-up, the external rotation was +54.5° (45–80°), internal rotation remained unchanged, and the gain in abduction and forward flexion averaged +150° (130–170°)					
Kokkalis et al. [6]	–	–	−30°	45°	90°	130°	12	17
Naoum et al. [25]	–	–	Improved by 52° (40–60°) at 6 months, but showed a gradual loss of improvement with time, reaching an overall improvement of 35° (25–45°) at last follow-up		Improved by 21° (15–45°) at 6 months and continued to improve right up to the last follow-up, at which there was an overall improvement of 31° (20–50°)		Improved by 0.58 points (0.4–0.9) at 6 months and continued to improve over time, reaching 0.6 points (0.45–0.95) at last follow-up	
Current study	–	–	−24°	+46°	85°	128°	11	17

*FU* follow-up, *PHHA* percentage of the humeral head anterior to the middle of the glenoid fossa, *External rotation* passive external rotation with the arm at the side, *Elevation* active elevation against gravity (approximated by compelling the child to reach for objects overhead)

## Discussion

Several surgical techniques to achieve shoulder alignment and function after internal rotation contracture of the shoulder joint following Erb’s palsy have been described [6]. In most procedures, the external rotation is obtained by tenotomy, lengthening, or release of the prime internal rotators of the shoulder—the subscapularis or pectoralis major muscles. Strecker et al. [9] studied 16 cases treated by the L’Episcopo–Sever technique [10, 11] and noted an average reduction in the active internal rotation of the shoulder joint 39 months after the operation. In a cadaveric study by Ferrari [12] and Harryman et al. [13], they concluded that the capsuloligamentous structures are the main restraints on external rotation. Although tendon transfer operations can improve the range of motion, these operations do not restore the normal glenohumeral joint alignment long term [14–16]. In a study by Van der Sluijs et al. [17] in which 19 patients underwent open reduction with subscapularis tendon lengthening, there was a significant increase in the Mallet score, but 42 % of their patients later developed an external rotation contracture which did not improve over time. Significant decreases in glenoid retroversion after open reduction with tendon lengthening or transfer were reported [15, 18]. The authors reported improvement after a subtotal subscapularis muscle, capsule, and tight ligament arthroscopic release procedure for internal rotation contracture of the shoulder joint due to Erb’s palsy with or without tendon transfer [4, 20–24]. In a study by Pearl et al. [22] in which they studied 33 children with a mean age of 3.7 years who were treated with arthroscopic release with or without latissimus dorsi tendon transfer, they reported improvements in passive external rotation of up to 45° in all but one patient after the arthroscopic release. Pearl et al. [22] concluded that, in children up to 3 years of age, arthroscopic release alone effectively restores near-normal passive external rotation and a centered glenohumeral joint at the time of surgery. The authors reported rapid remodeling of the glenoid after the arthroscopic shoulder joint reduction [20, 21, 24]. Kozin et al. [19] studied 44 children with Erb’s palsy and reported significant improvement in both clinical and radiologic outcomes of shoulder joint function after arthroscopic release with or without tendon transfer to restore glenohumeral joint alignment. Those successful and promising results are in good accord with the results of the current study, as all patients in the present series were 1–3 years old, which was the main age group targeted for the arthroscopic release alone procedure.

In summary, there are many advantages of the arthroscopic release procedure, as it allows more precise identification of the coracohumeral, superior glenohumeral, and middle glenohumeral ligaments. At the same time, as much release as required and dynamic assessment can be performed under arthroscopic control. Unlike open techniques, this procedure can be repeated for revision without much difficulty or scar tissue, and also it prevents permanent glenohumeral deformation, which is not always reversible. However, this approach does have disadvantages and complications. The first relates to the learning curve of the surgeon, as the number of pediatric shoulder arthroscopies performed is currently very small. Second, an excessive loss of internal rotation strength upon subscapularis insertional release may necessitate internal rotation humeral osteotomy. Third, the proximity of the axillary nerve puts it at risk of injury. Fourth, anterior instability may be created with the release of the associated glenohumeral ligaments. The current study also had some limitations, such as the lack of a control group and the fact that the operating surgeon also performed the follow-up evaluation.

## Conclusions

In conclusion, arthroscopic release alone yields good results in children aged from 1 to 3 years suffering from internal rotation contracture of the shoulder joint after Erb’s palsy. It has many advantages, as it addresses the primary pathology, associated with the capsuloligamentous structures. This procedure preserves the subscapularis and, at the same time, active internal rotation, so it prevents subsequent glenohumeral instability and later deformity.

## Electronic supplementary material

Below is the link to the electronic supplementary material.

Supplementary material 1 (MP4 39991 kb)
